# Dopamine Transporter Single-Photon Emission Computerized Tomography Supports Diagnosis of Akinetic Crisis of Parkinsonism and of Neuroleptic Malignant Syndrome

**DOI:** 10.1097/MD.0000000000000649

**Published:** 2015-04-03

**Authors:** G. Martino, M. Capasso, M. Nasuti, L. Bonanni, M. Onofrj, A. Thomas

**Affiliations:** From the Department of Radiology and Radiotherapy, Nuclear Medicine University G. d’Annunzio of Chieti-Pescara (GM, MN); Neurology Clinic, State Hospital (MC, LB, MO, AT); and Department of Neuroscience and Imaging and Aging Research Center, University G. d’Annunzio of Chieti-Pescara, Chieti, Italy (LB, MO, AT).

## Abstract

Akinetic crisis (AC) is akin to neuroleptic malignant syndrome (NMS) and is the most severe and possibly lethal complication of parkinsonism. Diagnosis is today based only on clinical assessments yet is often marred by concomitant precipitating factors. Our purpose is to evidence that AC and NMS can be reliably evidenced by FP/CIT single-photon emission computerized tomography (SPECT) performed during the crisis.

Prospective cohort evaluation in 6 patients. In 5 patients, affected by Parkinson disease or Lewy body dementia, the crisis was categorized as AC. One was diagnosed as having NMS because of exposure to risperidone. In all FP/CIT, SPECT was performed in the acute phase. SPECT was repeated 3 to 6 months after the acute event in 5 patients. Visual assessments and semiquantitative evaluations of binding potentials (BPs) were used. To exclude the interference of emergency treatments, FP/CIT BP was also evaluated in 4 patients currently treated with apomorphine.

During AC or NMS, BP values in caudate and putamen were reduced by 95% to 80%, to noise level with a nearly complete loss of striatum dopamine transporter-binding, corresponding to the “burst striatum” pattern. The follow-up re-evaluation in surviving patients showed a recovery of values to the range expected for Parkinsonisms of same disease duration. No binding effects of apomorphine were observed.

By showing the outstanding binding reduction, presynaptic dopamine transporter ligand can provide instrumental evidence of AC in Parkinsonism and NMS.

## INTRODUCTION

Around 0.3% of Parkinson disease (PD) patients per year suffer from a critical syndrome, akinetic crisis (AC) also termed malignant syndrome, acute akinesia, or Parkinson hyperpirexia syndrome,^1,2^ occurring concomitantly with infectious disease, surgery, trauma, or because of drug manipulation or withdrawal.^1,2^ AC is characterized by alterations of mental status, total akinesia with dysphagia, hyperthermia, dysautonomia and elevation of muscle enzymes, and by “absent response to rescue drugs or to dopaminergic drugs that adequately reduced symptoms before occurrence of the crisis.”^2^ AC symptoms are identical to symptoms of neuroleptic malignant syndrome (NMS) induced by typical and some atypical neuroleptics^2^ and by dopamine depleting drugs.^1^ A review of cause–effect relationship in NMS and AC^3^ underlined that there is no cause–effect ratio in NMS, at contrast with the serotoninergic syndrome, evidencing that AC is indistinguishable from NMS and suggested that both represent idiosyncratic severe complications induced by heterogeneous causes with (probably) common pathophysiology.^3^

AC and NMS diagnosis is often difficult because the concomitant precipitating events (eg, septicemia, endocarditis) may confound evaluation of core symptoms.^2^

Imaging of dopamine receptor might help understanding the mechanism of these acute syndromes, by evidencing postsynaptic or presynaptic binding alterations.^5,6^

Absent postsynaptic ligand-binding (iodobenzamide) was observed in a single NMS case,^4^ due to antidopaminergic drug administration. However, postsynaptic ligand-binding is extremely sensitive to interference of antidopaminergic (neuroleptics) treatment^7^; therefore, this finding was considered, by the authors of the report, expected and predictable.

Presynaptic, dopamine transporter (FP-beta CIT or ioflupane) binding instead is not modified by antidopaminergic drugs^8^ and the effect of dopaminergic drugs is considered also absent or minor^9^ despite some controversies.^10,11^ Therefore, presynaptic dopamine transporter-binding evaluations could be potentially useful for the assessment of both, AC or NMS. Two recent reports,^12,13^ describing each 2 cases of AC in hydrocephalus and in Parkinsonism, observed severe inhibition of ioflupane-binding. In our tertiary center^1,2^ for movement disorders emergencies (including AC or NMS)in prospective cohort evaluation, we could evaluate FP/CIT binding by single-photon emission computerized tomography (SPECT) in 6 patients, 5 with parkinsonism and definite AC and 1 with NMS.

To exclude interference of the rescue drug (apomorphine), we performed an ancillary SPECT study in 4 PD patients without AC, who were chronically treated with apomorphine at the same doses as the ones used for emergency treatment.^14^

## MATERIAL AND METHODS

### Patients With AC-NMS

During 2011 to 2014, 12 patients were addressed to our clinic because of AC in Parkinsonism, and 1 patient because of NMS. In 6 patients, we could evaluate FP/CIT SPECT scan during the acute phase of the crisis. Five of the 6 patients were addressed to our tertiary center because of AC, were affected by PD according to UKBBC,^15^ and were regularly followed in our clinic or in secondary clinics of the area. Table 1 summarizes clinical characteristics of patients and the precipitating factors. All patients/caregivers provided informed consent for therapy and neuroimaging, respecting the declaration of Helsinki.^16^

**TABLE 1:**
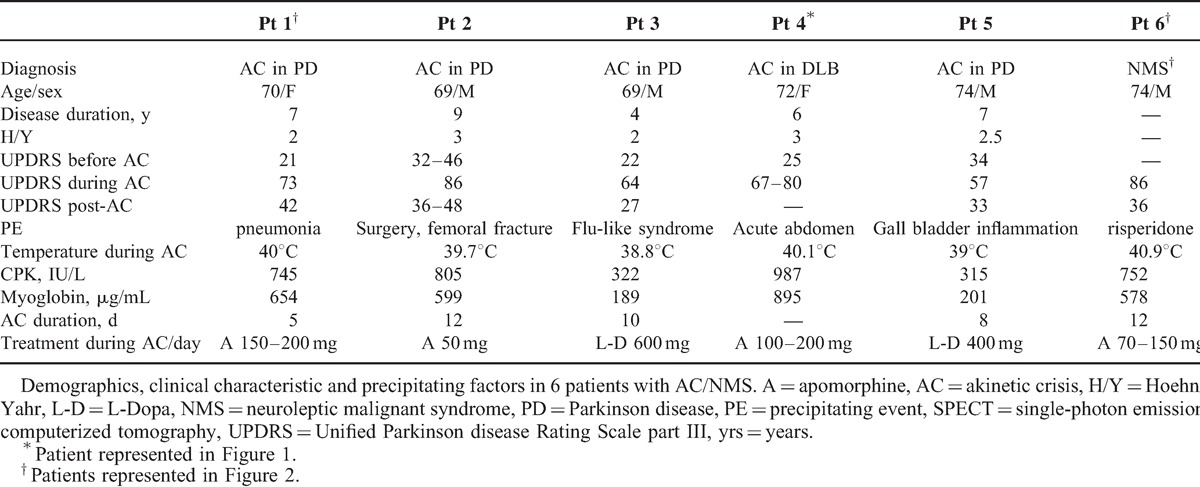
Demographics, Clinical Characteristics and Precipitating Factors

In 2 patients, AC was not related to withdrawal of dopaminergic drugs or administration of antidopaminergic drugs. In 1 patient, l-dopa withdrawal due to acute abdomen was the precipitating factor. One patient had NMS due to risperidone administration, but follow-up evidenced appearance of l-dopa responsive Parkinsonism despite no further exposition to antidopaminergic drugs occurred. Further details on methods are reported in online resource 1.

During AC, all patients were unresponsive to l-Dopa at the same drug regime that adequately corrected symptoms before the appearance of akinesia and were unresponsive to administration of dopaminergic rescue drug.^1,2^ Clinical signs were characterized by fever (>39°C), severe akinesia (UPDRS III 56.0 ± 9.1) dysphagia, labile blood pressure, confusional state and high serum levels of CPK (827.7 ± 124.8 IU/L), and myoglobuline (696.1 ± 139.9 ng/mL) (online resource 1). All vital parameters were constantly followed during the crisis.

During AC, all patients were treated with l-Dopa administered with nasogastric tube, according to prior drug regimens, and methyprednisolone 1 g/day, heparin, hydration. Four patients received continuous apomorfine infusion subcutaneously (rescue drug) with an electronic pump releasing 3.5 to 14 mg/h of apomorphine, for a total dose of 50 to 200 mg/24 h -day, for 2–4 days before SPECT acquisition. All Parkinsonian treatments were withdrawn 12 hours before ligand administration and were re-introduced only after imaging acquisition. Two patients received apomorphine immediately after SPECT acquisition. Five patients recovered completely or partially from AC in 5 to 12 days and returned to proper l-dopa regimens according to our treatment protocols and previous literature.^1,2,14^ The fourth patient died on day 22 because of ventricular tachyarrhythmia

### Effect of Apomophine in Non-AC-PD Patients

A possible effect of apomorphine on FP/CIT SPECT-binding potential (BP) was postulated in experimental studies on l-Dopa and dopamineagonists,^17–19^ although several studies did not evidence BP reduction induced by these drugs.^20,21^ To corroborate our findings, we tested the effect of apomorphine in 4 patients not affected by AC. Apomorphine, in continuous subcutaneous infusion, is currently used for the treatment of PD patients experiencing severe motor fluctuations, at the dose of 50 to 200 mg/day subcutaneously.^14,22^ Apomorphine is also indicated as a supportive, rescue treatment for AC and NMS^14,22^ at the same doses as used in chronic treatment.

Four patients, matched for age and disese duration with the 6 AC-NMS patients, all affected by PD according to UKBBC,^15^ all regularly treated with continuous subcutaneous apomorphine 100 to 200 mg/day (7–14 mg/h) because of severe motor fluctuations, underwent FP/CIT SPECT. All patients/caregivers provided informed consent for therapy and neuroimaging, respecting the declaration of Helsinki.^16^

**FIGURE 1:**
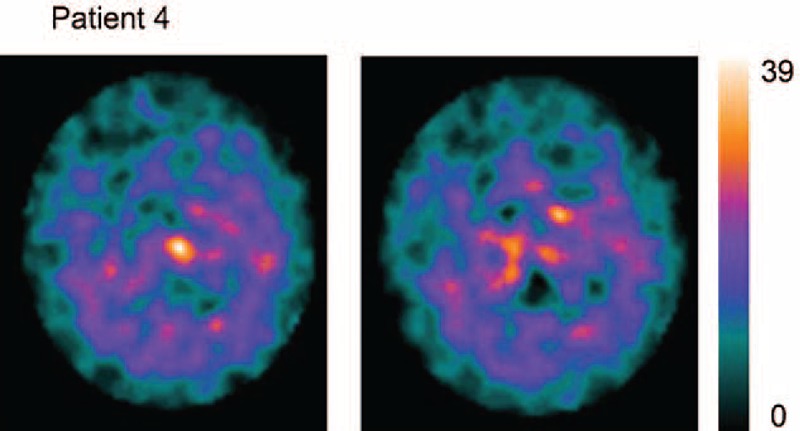
FP/CIT SPECT acquisition in patient 4, who died during AC. SPECT is plotted at the maximum saturation level: notice residual activity in areas corresponding to caudate, and the widespread distribution of the ligand, appearing with low levels in extrastiatal areas. Notice that the saturation level of the scale is 1/4 of scales reported in Figure 3, and a 1/5 to 1/4 of Figure 1. Binding uptake shows the typical pattern “burst striatum,” a severe bilateral reduction with almost no uptake in either the putamen or caudate with increased background uptake.^28,31^. AC = akinetic crisis, SPECT = single-photon emission computerized tomography.

The patients had undergone a first neuroimaging FP/CIT SPECT acquisition 5 ± 1 years before the last evaluation (Figure 1). All patients were also treated with l-Dopa. None of them presented with AC. All dopaminergic drugs were withdrawn 12 hours prior to scanning. Brain scanning was performed 4 hours after the infusion of 166.5 Bq of ^123^I-FP/Cit, performed according to SPECT scan methods and analyses as described in the following paragraph.

### DAT Acquisition and Analyses

**FIGURE 2:**
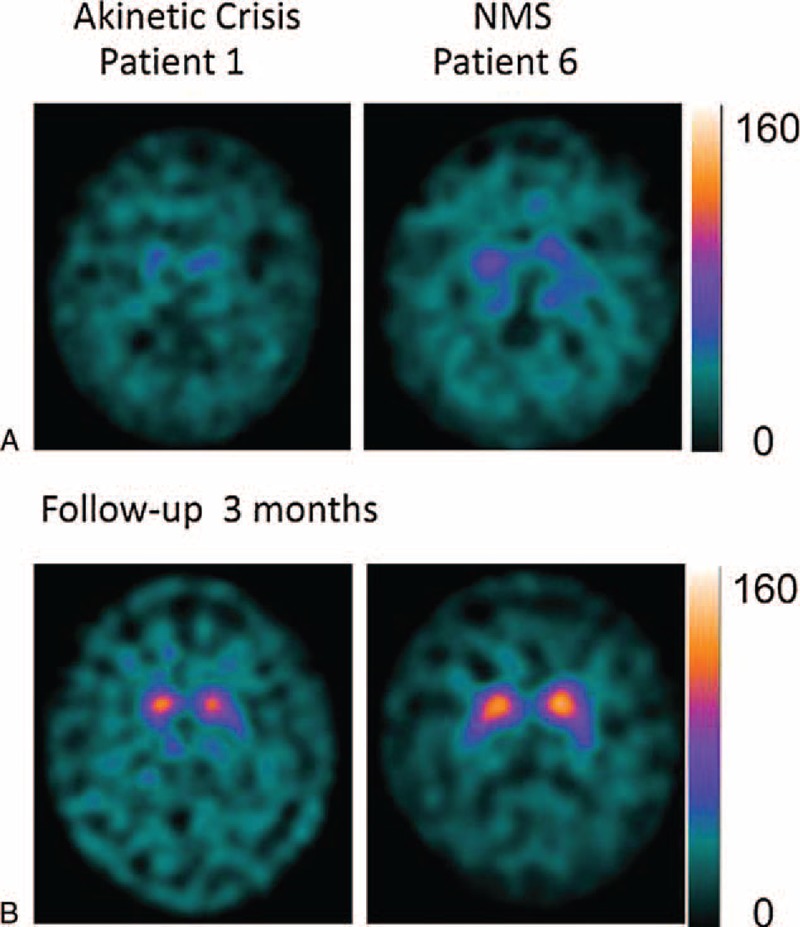
FP/CIT SPECT acquisition during AC/NMS, and after recovery, in patient 1 and 6. Notice almost complete disappearance of binding during the crises (“burst striatum”) and recovery (A), 3 months after the crises (B), with appearance of an “egg shape” pattern in patient 1 and a “mixed type” pattern in patient 6. Notice that saturation level scales are 4 times higher than the scales reported in figure 1. AC = akinetic crisis, NMS = neuroleptic malignant syndrome, SPECT = single-photon emission computerized tomography.

**FIGURE 3:**
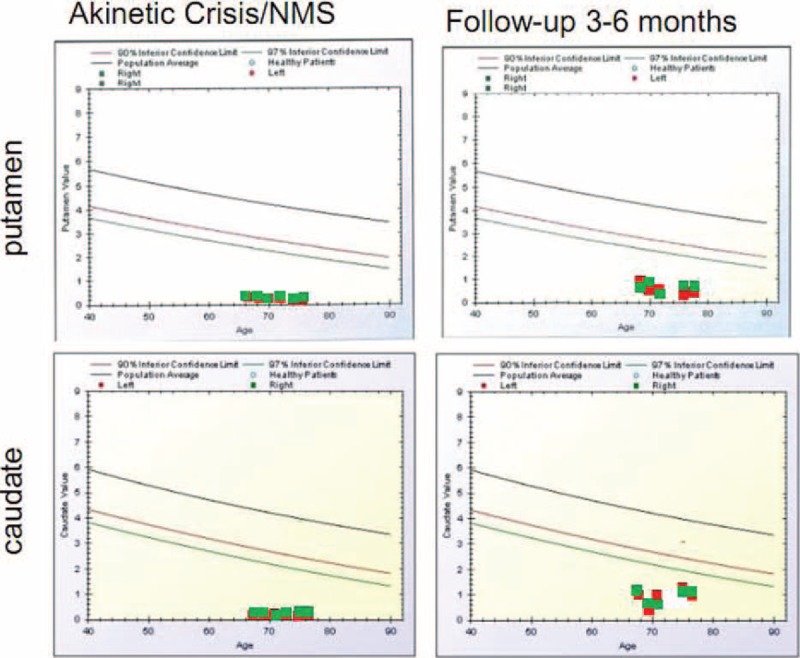
BasGan V2 analyses during AC/NMS in 6 patients and at the follow-up 3 to 6 months in the 5 surviving patients. Red dots indicate left putamen/caudate, and green dots indicate right putamen/caudate. AC = akinetic crisis, NMS = neuroleptic malignant syndrome.

123-I FP-CIT SPECTs were performed in all patients 48 to 72 hours after onset of the AC (Figures 2 and 3A) and 95 ± 2.8 days after recovery in survivors (Figure 3B), with GE, INFINIA double head gamma camera and evaluated according EANM procedure guidelines^23,24^ by Nuclear Medicine specialists blinded to diagnosis. All patients/caregivers gave informed consent before SPECT acquisition. This observational study received approval by our local ethical committee, according to the declaration of Helsinki and subsequent revisions.

**FIGURE 4:**
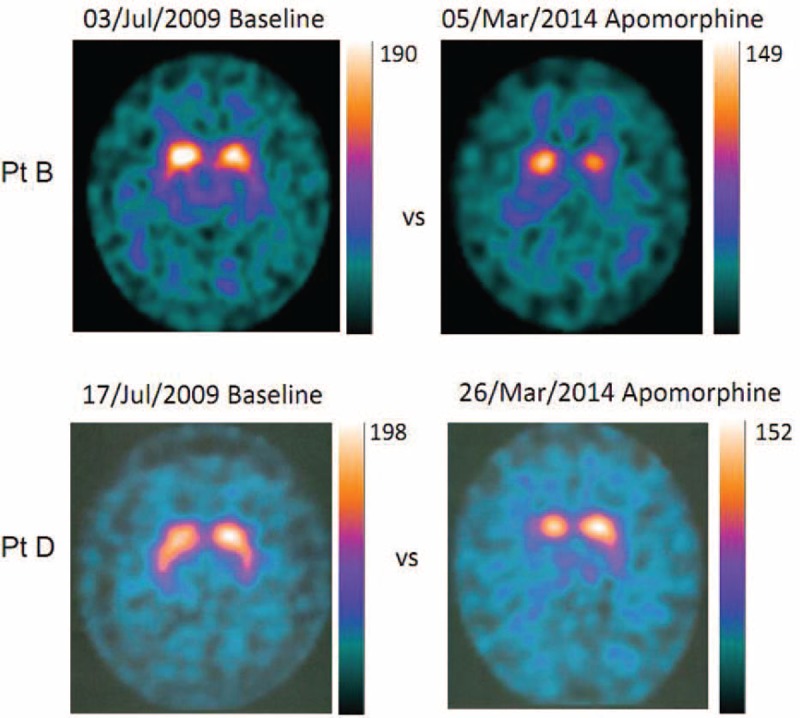
FP/CIT SPECT acquisition in two patients (PtB, PtD) chronically treated with apomorphine. Baseline SPECTs were acquired 6 years before, second acquisitions were obtained during chronic treatment. Notice that BP decay is in the range (Table 3) expected for the time lapsed from first acquisition, and the BP values are multiples of values (4–5 times) observed during AC (Figures 1 and 2). AC = akinetic crisis, BP = binding potential, SPECT = single-photon emission computerized tomography.

Brain SPECTs were acquired according to standard procedures.^24–26^ 166.5 Bq of 123-I-FP-CIT (DatSCAN Amerham Health, Amsterdam) was injected intravenously 60 min after administration of oral potassium iodide (Lugol solution) to block thyroid uptake of free radioactive iodide. Patients underwent imaging 4 hours after the injection, by means of a dedicated double head gamma camera equipped with ultrahigh-resolution collimators, matrix size 128 × 128, 120 frames, 40 s/frame, angular 3 degree, zoom 1.5. Slices (5.89-mm thick) were transversal, coronal, and sagittal canthomeatal-orientated Chang's^26^ correction with a coefficient of 0.11 cm. Two SPECT evaluation methods were used: semiquantitative analyses of cumulative dopamine transporter bindings^23^ by regions of interest (ROI) density evaluation (Table 2), a visual classification comparing color scale intensity of basal ganglia versus background/occipital areas and semiquantitative BasGanV2-assisted evaluation^27–29^ by an automated volume if interest (VOI) measurement (Figure 4), assessing total binding density in basal ganglia.

**TABLE 2:**
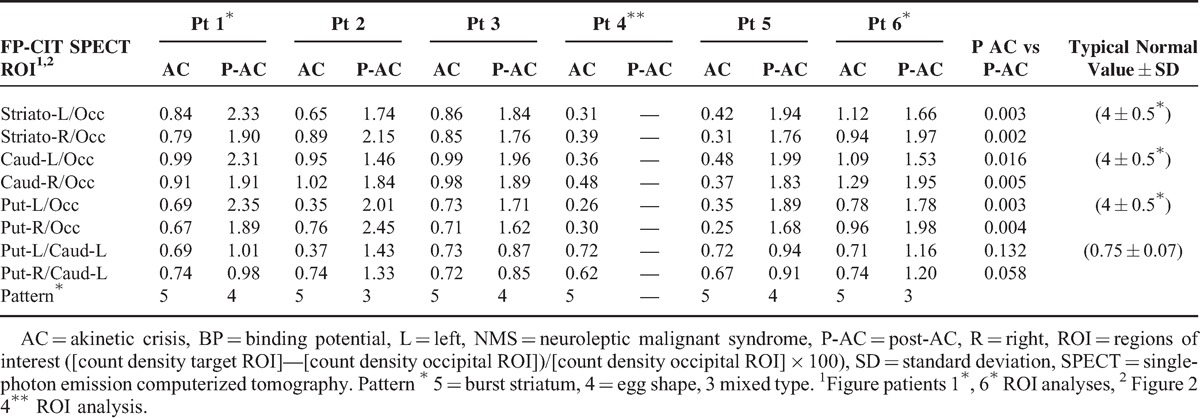
ROI Measurements in Patients With AC/NMS and Follow-up

For ROI analysis, 2 adjacent transaxial slices with the highest radiotracer uptake in the basal ganglia were summed (total thickness 11.78 mm) for semiquantitative analysis of caudate and putamen DAT-binding fixed ROIs (ROIs: 1 pixel 2.9 × 2.9 mm^2^) were drawn over the putamen, elliptical, 35 pixel, and the caudate, circular, 30 pixel, and an irregular occipital area of 265 pixel. The trace uptake was calculated, analyzing both sides, as: (count density target ROI) − (count density occipital ROI)/(count density occipital ROI) × 100.

For the second semiquantitative measurement, SPECT images were evaluated by a Basal Ganglia software (BasGanV2)^29,30^-assisted evaluation BasGanV2, a PC-based free software (http://aimn.it/struttura/gruppi/gs_neuro.php), which can be used for the analysis of DICOM images after transaxial reconstruction, a D operator-independent technique. The software draws automatically a 3D VOI) for each basal nucleus and provides background VOI; it determines the uptake/binding-potential values in the basal ganglia, and it compares the values obtained in each nucleus with reference to a database of 96 healthy subjects to determine age-adjusted values.^27,29^ The binding ratio for ^123^I-FP-CIT was calculated for putamen and caudate by ([mean counts of the target Tomean counts of background VOI]/[mean counts of the background VOI]). Wilcoxon signed-rank tests were used to assess statistical significance of BP recovery after AC-NMS in the 5 surviving patients. No statistical comparisons were performed in the ancillary study because of the time elapsed from first assessment and because the ancillary study was only designed to understand whether apomorphine-induced changes are comparable with alterations observed during AC-NMS.

The visual assessment method^32^ for 123-I-FP-CIT SPECT was also used, to provide simple descriptive evaluation. This method is based on the visual assessment grading system^32^ and categorizes tracer capture in 5 patterns.^28–30^ Pattern 1 is normal uptake, pattern 2 “eagle wing” shows discrete reduction prevalent in the mesial putamen, creating the shape of a wing (Figure 1, left, patient B), pattern 3 “mixed type” is asymmetric uptake reduction (Figure 1, left, patient D), the pattern 4, “egg shape” indicates no uptake in the putamen and preserved uptake in the caudate, resulting in an oval, egg shape of the caudate (Figure 3B, Figure 1, right, patient B), and the pattern 5 “burst striatum” indicates no uptake in the putamen and the striatum (Figures 2 and 3).

## RESULTS

### Patients During and After AC

Table 1 summarizes the characteristics of the 5 PD/DLB patients affected by AC and of 1 patient affected by NMS. No evidence of Parkinsonism was reported in patient 6 before the occurrence of NMS due to risperidone administration. During NMS, the patient presented with severe akinesia of the same severity as akinesia in PD/DLB patients, in which the precipiting causes were infections and bone trauma. Despite recovery, this patient at the 6-month follow-up presented a bilateral Parkinsonism (Hahn/Yahr stage 2) with an UPDRS score of 16.

During AC, FP/CIT scans showed in all patients reduced/absent uptake in the putamen and severely reduced or no uptake in the caudate of the patients. The visual analysis of SPECT acquisition evidenced aspects of “burst striatum” pattern 5^28,31^ in all patients. Figure 2 shows FP/CIT acquisition plotted at the maximum saturation level, in patient 4, who died 2 weeks after onset of symptoms. In the 4 PD patients who recovered from AC and in the patient with NMS, a second SPECT acquisition performed 3 to 6 months after recovery showed recovery of FP/CIT BP mostly evident in the caudate, same as expected for Parkinsonism with the same UPDRS score and disease duration, with pattern 3 “mixed type” and pattern 4 “egg shape” of visual categorization.

Figure 3 shows examples of FP/CIT SPECT acquisitions in 1 PD patient with AC and in the patient with NMS during the crisis and 3 to 6 months after recovery. Figure 4 shows the BasGanV2 VOIs plots from putamen and caudate in the 6 patients during AC/NMS and the follow-up at 3 to 6 months in the 5 surviving patients.^27,29^

The ROIs semiquantitative analysis is reported in Table 2. As shown in Table 2, during AC or NMS, BP was reduced to less than 1/6 to 1/10 of the age-matched control mean values. In recovering patients, BP increased in the caudate by 250% to 500% and in the putamen by 230% to 650% in comparison with acquisition during the crisis. Wilcoxon signed-rank tests evidenced that BP recovery in caudate areas was significant at *P* < 0.01, whereas recovery in putamen was significant at *P* < 0.05.

### Apomorphine Effect Assessment in Non-AC Patients

**FIGURE 5:**
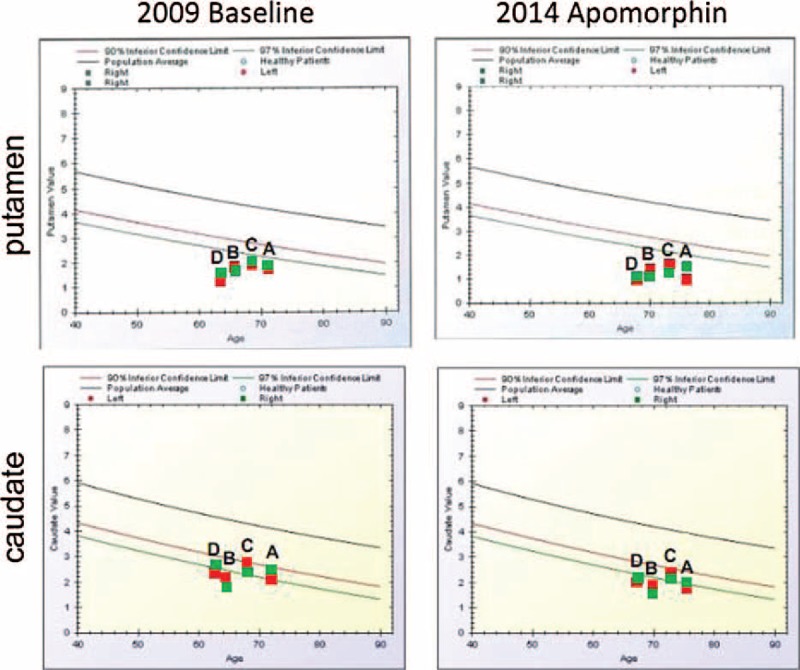
BasGan V2 analyses in 4 patients at baseline and 5 ± 1 years after during chronic treatment with apomorphine. Red dots indicate left putamen/caudate, and green dots indicate right putamen/caudate.

Figure 1 shows examples of FP/CIT SPECT acquisition in 2 patients who were regularly treated with apomorphine. For a comparison, SPECT acquisitions obtained several years before, at onset of motor fluctuations, are matched with SPECT acquisitions obtained during apomorphine treatment. No aspects of “burst striatum” were observed. FP/CIT SPECT BP during apomorphine treatment was in the range expected from PD patients of the same disease duration and severity.^33–35^ According to literature, presenting only ROI semiquantitative measurements, in PD patients, BP decay is by an annual rate of 5% to 10% in the putamen and by 3% to 6% in the caudate on an average.^28–36^Table 3 shows comparisons of baseline measurements and actual measurements during apomorphine treatment, and a comparison with the decay expected from literature data, in the time elapsed from baseline to acquisition during apomorphine treatment. The VOIs’ measurements in Figure 5 and the ROIs’ measurements reported in Table 3 show that the values measured in the 4 patients corresponded to a decay of <5% year for all patients. The BP values during apomorphine treatment were 2.5 to 7.5 times higher than in the AC-PD/NMS patients.

**TABLE 3:**
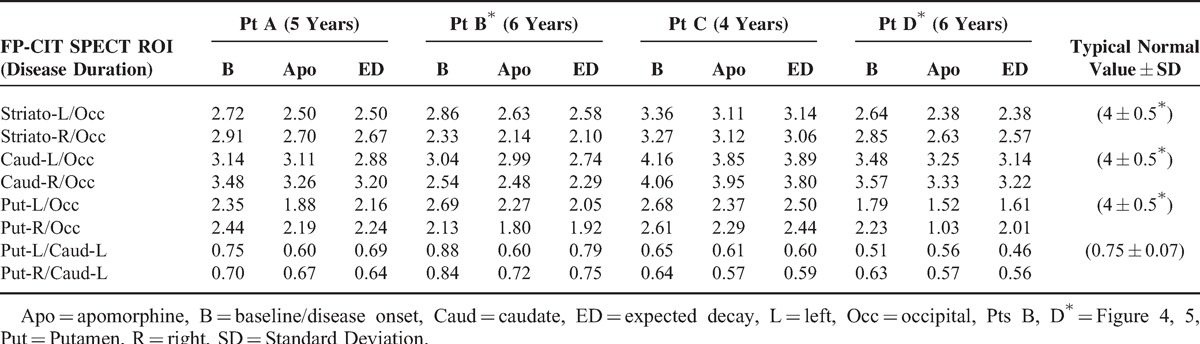
ROI Measurements in Patients Without AC at Onset of Severe Symptoms With Fluctuation and 5 ± 1 years After, During Chronic Apomorphine Administration

## DISCUSSION

Our findings offer the unprecedented evidence that FP/CIT uptake is absent/extremely reduced in AC and NMS, and that instrumental assessment with SPECT might help the identification of these challenging and possibly lethal syndromes.

During AC and NMS, FP/CIT SPECT showed in all patients reduced/absent uptake in the putamen bilaterally, yet more interesting was the finding of extremely reduced activity in the caudate nucleus (Figures 1 and 2), resulting in the feature of “burst striatum” with disappearance of the oval-shaped images corresponding to caudate. Semiquantitative evaluations showed uptake reductions to noise level of scales (Figure 3, Table 2).

During follow-up, repeated scans showed increment of FP/CIT uptake, with resumption of the typical patterns observed in progression of Parkinsonism.^32–35^ These findings suggest that during AC or NMS, dopamine presynaptic transporter activity is inefficient and that this dysfunction may recover during follow-up in surviving patients. Two previous case reports,^12,13^ describing 2 patients each, showed also severe suppression of FP/CIT uptake in patients with AC. Only 2 patients survived the crisis, in one recovery of BP was observed, in the other instead, despite clinical recovery, no improvement in FP/CIT uptake was reported, even though SPECT figures showed a “burst striatum” pattern during AC and an “oval shape” (caudate activity) after recovery. We suggest that the utilization of 2 semiquantitative evaluations and of the visual categorization methods, in our study, gave proper evidence of functional recovery, by allowing to overcome difficulties due to low level of uptakes in Parkinsonian conditions.

During the progressive course of Parkinsonism, FP/CIT SPECT alterations appear because of reduction of BP. These alterations are mostly evidenced in the putamen, initially in the mesial part and in the more severe cases in the entire putamen, with the appearance of “eagle wing” and “egg shape” patterns of visual assessment categorization.^27,29,32^ Uptake in the caudate is relatively preserved (eg, “egg shape” is due to the preservation of the oval, caudate, shape and disappearance of the “wing” representing the putamen). The “burst striatum” pattern designs disappearance of uptake in the caudate areas. This pattern appears in severe Parkinsonism, and as shown in our results during AC and NMS.^27,29^

For this reason, we suggest that extreme reduction/disappearance of caudate activity might constitute a tentative operational indication for AC identification. Visual assessment of a “burst striatum” pattern, with disappearance of caudate uptake in patients with moderately severe Parkinsonism before the crisis, could identify the presence of AC, and could evidence NMS.

Semiquantitative evaluations (Table 2, Figure 3) suggest that reduction of caudate FP/CIT BP to less than 1/5 of the value corresponding to the 97% inferior confidence limit for age-matched control could also suggest a diagnosis of AC/NMS. To make, of the proposed SPECT instrumental assessment of AC-NMS, a definite diagnostic tool, our study fosters further studies correlating PD severity duration with SPECT findings and establishing unequivocal measurement methods. At present, only 4 studies are available.^4,7,12,13^

The attempt to understand the mechanism underlying the observed dopamine transporter suppression during AC and NMS requires further considerations.

First, as AC and NMS are life-threatening conditions, urge to treat (with putative rescue drugs) made it impossible to withdraw treatments, until ligands were available for SPECT acquisition, in 4 patients. Thus, it might be argued that the observed alterations are due to drug effects, that is, effect of risperidone in the NMS case and effect of l-Dopa or apomorphine in the NMS and AC cases. Despite several previous studies evidenced that dopamine agonists (DAs), l-dopa, and neuroleptics do not alter dopamine transporter-binding,^4,8,9^ fewer studies challenged these evidences.^5,10,11^ Theoretical considerations suggest that DAs may upregulate, whereas l-dopa may downregulate dopamine transporter-binding.^31,36^ Other clinical experimental studies showed instead uptake reductions induced by l-Dopa or DAs, in the range of 3% to 22%.^11^

Several of our findings challenge the treatment effect hypothesis: first, reductions of presynaptic dopamine transporter binding to noise level during AC, increments by 230% to 650% (caudate) in recovering patients, as observed in our patients, are of a magnitude incompatible with predictions resulting from studies showing drugs effects on DAT-binding.^10,11,31,36^

Second, 2 of the patients of the present case series were not treated with apomorphine. The patients, with AC and FP/CIT SPECT uptake reduction in the 2 previous case reports,^12,13^ were not treated.

Third, the l-Dopa doses administered to our patients were in the range commonly used in PD patients undergoing SPECT assessment. Apomorhine doses were also in the standard treatment range^1,2,14^ and apomorphine was withdrawn from the night before assessment. Apomorphine has a half-life of 45 min; therefore, it is unlikely that the finding, in the 4 AC patients who received apomorphine as a rescue drug, is dependent on drug interactions, as apomorphine and all other treatments were withdrawn 12 hours before SPECT acquisition. According to half life^22,37^ apomorphine should have disappeared at the time of SPECT acquisition.

Fourth, in the ancillary SPECT study performed in 4 Parkinsonian patients, age- and disease duration-matched, who were not affected by AC but who were regularly treated with apomorphine, the FP/CIT BP was evidently above the levels observed in AC and NMS patients; in the range expected for Parkinsonisms with similar progression,^32–35^ no “burst striatum” patterns were observed.

As an alternative hypothesis to drug effect, we suggest that the explanation for our finding could be that AC is due to altered energetic state, same as NMS, wherein mitochondrial complex I activity inhibition was observed.^38,39^

According to this hypothesis, a recent study^40^ evidenced that AC is more frequent in monogenic PD due to mitochondrial dysfunction than in “idiopathic” parkinsonism's. In POLG mutations, a pathology due to mitochondrial DNA mutations^41^ FP/CIT BP is severely reduced, even in absence of Parkinsonism.^42^

Mitochondrial dysfunction and altered energetic state would alter the proton adenosine triphosphatase pump needed for dopamine transporter activity,^36^ leading to dopamine transporter suppression, and would also alter synaptic dopamine release. Because of the reduced dopamine release, due to the energetic failure, also dopamine transporter expression would furtherly be depressed, in the compensatory attempt to maintain dopamine concentration in the synapse.^36^ This hypothesis may explain the unprecedented finding, observed in the present study, of the almost complete suppression of FP-CIT-binding during AC and NMS.

Therefore, our conclusion is that FP-CIT SPECT, by evidencing severe BP reduction with the “burst striatum” pattern, in both putamen and caudate can be used to assess both AC and NMS.

## References

[1] Thomas A Onofrj M Akinetic crisis, acute akinesia, neuroleptic malignant-like syndrome, Parkinsonism-hyperpyrexia syndrome, and malignant syndrome are the same entity and are often independent of treatment withdrawal. *Mov Discord* 2000; 20:1671. 10.1002/mds.20689 16149099

[2] Onofrj M Thomas A Acute akinesia in Parkinson disease. *Neurology* 2005; 64:1162–1169. 15824341 10.1212/01.WNL.0000157058.17871.7B

[3] Margetić B Aukst-Margetić B Neuroleptic malignant syndrome and its controversies. *Pharmacoepidemiol Drug Saf* 2010; 19:429–435. 20306454 10.1002/pds.1937

[4] Jauss M Krack P Franz M Imaging of dopamine receptors with (123I)iodobenzamide single-photon emission-computed tomography in neuroleptic malignant syndrome. *Mov Disord* 1996; 11:726–728. 8914102 10.1002/mds.870110621

[5] Klein JC Eggers C Kalbe E Neurotransmitter changes in dementia with Lewy bodies and Parkinson disease dementia in vivo. *Neurology* 2010; 74:885–892. 20181924 10.1212/WNL.0b013e3181d55f61

[6] Eggers C Hilker R Burghaus L High resolution positron emission tomography demonstrates basal ganglia dysfunction in early Parkinson's disease. *J Neurol Sci* 2009; 276:27–30. 18835490 10.1016/j.jns.2008.08.029

[7] Lorberboym M Treves TA Melamed E (123I)-FP/CIT SPECT imaging for distinguishing drug-induced parkinsonism from Parkinson's disease. *Mov Disord* 2006; 21:510–514. 16250023 10.1002/mds.20748

[8] Ahlskog JE Uitti RJ O’Connor MK The effect of dopamine agonist therapy on dopamine transporter imaging in Parkinson's disease. *Mov Disord* 1999; 14:940–946. 10584667 10.1002/1531-8257(199911)14:6<940::aid-mds1005>3.0.co;2-y

[9] Schillaci O Pierantozzi M Filippi L The effect of levodopa therapy on dopamine transporter SPECT imaging with(123)I-FP-CIT in patients with Parkinson's disease. *Eur J Nucl Med Mol Imaging* 2005; 32:1452–1456. 16151764 10.1007/s00259-005-1922-9

[10] Sossi V Dinelle K Schulzer M Levodopa and pramipexole effects on presynaptic dopamine PET markers and estimated dopamine release. *Eur J Nucl Med Mol Imaging* 2010; 37:2364–2370. 20697890 10.1007/s00259-010-1581-3

[11] Guttman M Stewart D Hussey D Influence of L-dopa and pramipexole on striatal dopamine transporter in early PD. *Neurology* 2001; 56:1559–1564. 11402115 10.1212/wnl.56.11.1559

[12] Jussen D Sprung C Buchert R Hydrocephalus-induced neuroleptic malignant-like syndrome with reduced dopamine transporters. *J Neurol* 2013; 260:2182–2214. 23835636 10.1007/s00415-013-7026-8

[13] Kaasinen V Joutsa J Noponen T Akinetic crisis in Parkinson's disease is associated with a severe loss of striatal dopamine transporter function: a report of two cases. *Case Rep Neurol* 2014; 6:275–280. 25566059 10.1159/000369448 PMC4280458

[14] Deutsche Neurologische Gesellschaft Leitlinien fuer Parkinson Syndrome (2012): Äquivalenzdosen Table. 9. 4 and Table. 9. 5.

[15] Hughes AJ Daniel SE Kilford L Accuracy of clinical diagnosis of idiopathic Parkinson's disease: a clinico-pathological study of 100 cases. *J Neurol Neurosurg Psychiatry* 1992; 55:181–184. 1564476 10.1136/jnnp.55.3.181 PMC1014720

[16] WMA Declaration of Helsinki—Ethical Principles for Medical Research Involving Human Subjects Adopted by the 18th WMA General Assembly, Helsinki, Finland, June 1964 and amended by the: 64th WMA General Assembly, Fortaleza, Brazil, October 2013.

[17] Zhang Z Andersen AH Avison MJ Functional MRI of apomorphine activation of the basal ganglia in awake rhesus monkeys. *Brain Res* 2000; 852:290–296. 10678755 10.1016/s0006-8993(99)02243-x

[18] Parkinson Study Group. Dopamine transporter brain imaging to assess the effects of pramipexole vs levodopa on Parkinson disease progression. *JAMA* 2002; 287:1653–1661. 11926889 10.1001/jama.287.13.1653

[19] Passamonti L Salsone M Toschi N Dopamine-transporter levels drive striatal responses to apomorphine in Parkinson's disease. *Brain Behav* 2013; 3:249–262. 23785657 10.1002/brb3.115 PMC3683285

[20] Antonini A Schwarz J Oertel WH (11C)raclopride and positron emission tomography in previously untreated patients with Parkinson's disease: Influence of L-dopa and lisuride therapy on striatal dopamine D2-receptors. *Neurology* 1994; 44:1325–1329. 8035939 10.1212/wnl.44.7.1325

[21] Ahlskog JE Slowing Parkinson's disease progression: recent dopamine agonist trials. *Neurology* 2003; 60:381–389. 12580184 10.1212/01.wnl.0000044047.58984.2f

[22] Bowron A Practical considerations in the use of apomorphine injectable. *Neurology* 2004; 62 (6 suppl 4):S32–S36. 15037670 10.1212/wnl.62.6_suppl_4.s32

[23] Van Laere K Varrone A Booij J EANM procedure guidelines for brain neurotransmission SPECT/PET using dopamine D2 receptor ligands, version 2. *Eur J Nucl Med Mol Imaging* 2010; 37:434–442. 19838704 10.1007/s00259-009-1265-z

[24] Darcourt J Booij J Tatsch K EANM procedure guidelines for brain neurotransmission SPECT using (123)I-labelled dopamine transporter ligands, version 2. *Eur J Nucl Med* 2010; 37:443–450. 10.1007/s00259-009-1267-x 19838702

[25] Booij J Hemelaar TG Speelman JD One-day protocol for imaging of the nigro-striatal dopaminergic pathway in Parkinson's disease by (123I)FP-CIT SPECT J. *Nucl Med* 1999; 40:753–761. 10319746

[26] Chang LT A method for attenuation correction in radionuclide computed tomography. *IEEE Trans Nucl Sci* 1978; 25:638–643.

[27] Calvini P Rodriguez G Inguglia F The basal ganglia matching tools package for striatal uptake semi-quantification: description and validation. *Eur J Nucl Med Mol Imaging* 2007; 34:1240–1253. 17287959 10.1007/s00259-006-0357-2

[28] Kahraman D Eggers C Schicha H Visual assessment of dopaminergic degeneration pattern in 123I-FP-CIT SPECT differentiates patients with atypical parkinsonian syndromes and idiopathic Parkinson's disease. *J Neurol* 2012; 259:251–260. 21750954 10.1007/s00415-011-6163-1

[29] Skanjeti A Angusti T Iudicello M Assessing the accuracy and reproducibility of computer-assisted analysis of (123) I-FP-CIT SPECT using BasGan (V2). *J Neuroimaging* 2014; 24:257–265. 23323544 10.1111/jon.12008

[30] Davidsson A Georgiopoulos C Dizdar N Comparison between visual assessment of dopaminergic degeneration pattern and semi-quantitative ratio calculations in patients with Parkinson's disease and Atypical Parkinsonian syndromes using DaTSCAN(®) SPECT. *Ann Nucl Med* 2014; 28:851–859. 24997753 10.1007/s12149-014-0878-x

[31] Nurmi E Bergman J Eskola O Reproducibility and effect of levodopa on dopamine transporter function measurements: a (18F)CFT PET study. *J Cereb Blood Flow Metab* 2000; 20:1604–1609. 11083235 10.1097/00004647-200011000-00010

[32] Benamer HT Patterson J Wyper DJ Correlation of Parkinson's disease severity and duration with 123I-FP-CIT SPECT striatal uptake. *Mov Disord* 2000; 15:692–698. 10928580 10.1002/1531-8257(200007)15:4<692::aid-mds1014>3.0.co;2-v

[33] Pirker W Holler I Gerschlager W Measuring the rate of progression of Parkinson's disease over a 5-year period with beta-CIT SPECT. *Mov Disord* 2003; 18:1266–1272. 14639666 10.1002/mds.10531

[34] Schwarz J Storch A Koch W Loss of dopamine transporter binding in Parkinson's disease follows a single exponential rather than linear decline. *J Nucl Med* 2004; 45:1694–1697. 15471835

[35] Colloby SJ Williams ED Burn DJ Progression of dopaminergic degeneration in dementia with Lewy bodies and Parkinson's disease with and without dementia assessed using 123I-FP-CIT SPECT. *Eur J Nucl Med Mol Imaging* 2005; 32:1176–1185. 15931516 10.1007/s00259-005-1830-z

[36] Benarroch EE Monoamine transporters: structure, regulation, and clinical implications. *Neurology* 2013; 81:761–768. 23902707 10.1212/WNL.0b013e3182a1ab4a

[37] LeWitt PA Subcutaneously administered apomorphine: pharmacokinetics and metabolism. *Neurology* 2004; 62 (6 suppl 4):S8–S11. 15037665 10.1212/wnl.62.6_suppl_4.s8

[38] Burkhardt C Kelly JP Lim YH Neuroleptic medications inhibit complex I of the electron transport chain. *Ann Neurol* 1993; 33:512–517. 8098932 10.1002/ana.410330516

[39] Maurer I Moller HJ Inhibition of complex I by neuroleptics in normal human brain cortex parallels the extrapyramidal toxicity of neuroleptics. *Mol Cell Biochem* 1997; 174:255–259. 9309697

[40] Bonanni L Onofrj M Valente EM Recurrent and fatal akinetic crisis in genetic-mitochondrial parkinsonisms. *Eur J Neurol* 2014; 21:1242–1246. 24471704 10.1111/ene.12364

[41] Invernizzi F Varanese S Thomas A Two novel POLG1 mutations in a patient with progressive external ophthalmoplegia, levodopa-responsive pseudo-orthostatic tremor and parkinsonism. *Neuromuscul Disord* 2008; 18:460–464. 18502641 10.1016/j.nmd.2008.04.005

[42] Tzoulis C Tran GT Schwarzlmüller T Severe nigrostriatal degeneration without clinical parkinsonism in patients with polymerase gamma mutations. *Brain* 2013; 136 (Pt 8):2393–2404. 23625061 10.1093/brain/awt103

